# Children with Secondary Care Episodes for Otitis Media Have Poor Literacy and Numeracy Outcomes: A Data Linkage Study

**DOI:** 10.3390/ijerph182010822

**Published:** 2021-10-15

**Authors:** Megan F. Bell, Fernando Lima, Deborah Lehmann, Rebecca Glauert, Hannah C. Moore, Christopher G. Brennan-Jones

**Affiliations:** 1 Telethon Kids Institute, University of Western Australia, P.O. Box 855, West Perth, WA 6872, Australia; fernando.lima@unisa.edu.au (F.L.); rebecca.glauert@uwa.edu.au (R.G.); 2 Wesfarmers Centre of Vaccines and Infectious Diseases, Telethon Kids Institute, University of Western Australia, P.O. Box 855, West Perth, WA 6872, Australia; deborah.lehmann@telethonkids.org.au (D.L.); hannah.moore@telethonkids.org.au (H.C.M.); chris.brennan-jones@telethonkids.org.au (C.G.B.-J.); 3 Division of Paediatrics, Faculty of Health and Medical Sciences (M561), University of Western Australia, 35 Stirling Highway, Crawley, WA 6009, Australia; 4 Perth Children’s Hospital, Child and Adolescent Health Service, Nedlands, WA 6009, Australia; 5 Faculty of Health Sciences, Curtin University, Bentley, WA 6102, Australia

**Keywords:** otitis media, literacy, numeracy, linked administrative data

## Abstract

We examined the association between otitis media (OM) and educational attainment in a retrospective population cohort of Western Australian children who participated in the grade 3 National Assessment Program—Literacy and Numeracy in 2012 (*N* = 19,262). Literacy and numeracy scores were linked to administrative hospital and emergency department data to identify secondary care episodes for OM. Results of multivariate multilevel models showed that children with OM episodes had increased odds of poor performance on literacy and numeracy tests, compared to children without OM episodes (46–79% increase in odds for Aboriginal children; 20–31% increase in odds for non-Aboriginal children). There were no significant effects found for age at the first episode, nor for OM episode frequency (all *p*s > 0.05). Regardless of the timing or frequency of episodes, children with OM episodes are at risk of poor literacy and numeracy attainment. Aboriginal children with OM appeared to be particularly at risk of poor literacy and numeracy achievement. Intervention to reduce the prevalence of otitis media in young children, and early treatment of OM, are important for limiting the negative effects on academic outcomes.

## 1. Introduction

Otitis media (OM), a spectrum of inflammatory conditions involving the middle ear, is one of the most common conditions affecting young children [1]. Australian Aboriginal and/or Torres Strait Islander children (hereafter, respectfully referred to as Aboriginal) are particularly susceptible to OM [2,3], with the highest reported rates of OM globally [4]. Australian Aboriginal children are five times more likely to be diagnosed with OM in primary care settings than non-Aboriginal children [5], and 10 times more likely to be hospitalized for OM [3]. OM is the most common cause of temporary mild-moderate conductive unilateral or bilateral hearing loss in children [1]. Recurrent or persistent episodes of OM often result in long periods of poor hearing (hearing loss of 15–40 dB) or complications that may result in permanent hearing loss [6]. This hearing loss results in an inconsistent auditory stimulus, which may negatively affect early development of speech and language [7,8,9], and thus have adverse consequences for children’s learning and social development [10].

The peak prevalence of OM occurs between 6 to 18 months of age [1], which coincides with a period of rapid development for children. Recurrent or persistent OM occurring in the first years of life may thus be particularly detrimental to long-term outcomes. Previous research has found an association between OM and poor school readiness [11]; academic, speech, and language difficulties [12,13,14,15]; and emotional, attention, and behavior problems [12,16,17]. However, some researchers argue that these associations are explained by confounding variables common to both OM incidence and poor academic attainment [18], including preterm birth, socioeconomic disadvantage, and poor school attendance. The contradiction in the literature is related to methodological limitations. Many studies do not control for probable confounding variables, utilize small samples, have high attrition rates, and primarily rely on retrospective parent reports, which can be associated with recall and reporting bias. Few studies utilize linked administrative data; this methodology is prospective, provides large samples, captures clinical health information, and has minimal loss to follow-up.

We use linked data to examine the association between secondary care episodes for OM in childhood (0–9 years) and performance on standardized tests of literacy and numeracy in grade 3, controlling for multiple potential confounding factors. We hypothesize that children who have secondary care episodes for OM will have low attainment on grade 3 literacy and numeracy tests, and that this association will be stronger for children with a first episode prior to 3 years of age, and those who have repeated OM episodes.

## 2. Materials and Methods

### 2.1. Setting and Study Population

This is a cohort study in which we examined the association between secondary care episodes for OM and scores on standardized numeracy and literacy assessments for children in grade 3. The study was conducted in Western Australia (WA), which has an area of 2.5 million km^2^ and a population of 2.6 million, 79% of whom reside in the capital of Perth. Health services in WA are managed by public and private healthcare providers and there are >80 hospitals. All WA residents are eligible for free care at public hospitals.

The study population included children born in WA during July 2003 to June 2004 who subsequently participated in the grade 3 National Assessment Program—Literacy and Numeracy (NAPLAN) in 2012 (*N* = 20,479). Exclusion criteria were (1) missing all NAPLAN scores (*n* = 847); (2) multiple births, with one child from each birth retained by random selection (excluded *n* = 296); and (3) diagnosed with intellectual disability as recorded in the IDEA (Intellectual Disabilities Exploring Answers) database (*n* = 74). The final sample included 19,262 children who were aged 7–9 years at the end of the study period (May 2012). Prospectively collected hospital and emergency department records for all study children were extracted (see details below) for identification of children with secondary care episodes for OM.

### 2.2. Data Sources

Data were provided by the WA Departments of Health and Education, the Commonwealth Department of Education, and the WA Register of Developmental Anomalies (WARDA). Datasets were linked by the WA Data Linkage Branch by probabilistic matching of identifiers common to the sets of records (e.g., name, address) and clerical review [19]. Only de-identified records were provided to researchers.

### 2.3. Otitis Media Episodes

Data from secondary care episodes were used to identify children with diagnoses of OM. Secondary care episodes were defined as admissions to hospital or presentations to an emergency department, sourced from the Hospital Morbidity Data Collection (HMDC; records all WA public and private hospital separations), and the Emergency Department Data Collection (EDDC; records presentations to WA public emergency departments), respectively. Secondary care episodes where a child received a diagnosis of OM or where symptoms indicative of OM were recorded were classified as ‘OM episodes.’ Children who did not have a secondary care OM episode were included in the ‘no OM episodes’ group (noting that these children may have been diagnosed with OM in a primary care setting).

In this study, OM refers to a broad range of presentations for middle ear infections and their sequelae, including acute OM, OM with effusion, conductive hearing loss, chronically discharging perforations, cholesteatoma, and mastoiditis. Diagnoses were coded by trained coders using the International Classification of Diseases Tenth Revision, Australian Modification (ICD-10-AM) [20]. As per Westphal et al. [3], ICD-10-AM codes H65-67, H70-75, H90.0-H90.2, H90.6-H90.8, H92, and/or H95 were used to identify OM episodes in any of the HMDC primary or additional diagnosis fields.

In the EDDC, the same ICD-10-AM codes were used to identify all principal diagnoses of OM. As per Barnes et al. [21], other EDDC variables were used to identify OM episodes when principal diagnosis was not recorded. First, symptom codes for ‘ear discharge’ (FC000 and FGB00) were considered to indicate OM presentations. If both principal diagnosis and symptom codes were missing, ‘diagnosis at discharge’ and ‘presenting complaints’ free text variables were used. Free text of, for example, ‘left otitis media’, was considered an OM episode. OM episodes were identified for all children from month of birth until the end of the study period (May 2012). Appendix A details the number of OM episodes identified from each data source and for each ICD-10-AM code.

### 2.4. Academic Outcomes

Academic test scores were obtained from the 2012 NAPLAN, when the cohort children were in grade 3. The NAPLAN is an annual national assessment of children’s academic performance in numeracy, reading, writing, spelling, and grammar and punctuation. This study focused on the numeracy, reading, and writing tests. All Australian students are expected to undertake the NAPLAN. In 2012, approximately 95% of WA students enrolled in grade 3 participated [22]. Tests are conducted in schools over three consecutive days in May. Children are given 40–45 min for each testing session. Questions are multiple-choice or short answer and are curriculum-based. Children’s scores on each test are compared against national benchmarks according to the age at which they complete the test. A child scoring at or above the ‘national minimum standards’ has demonstrated the basic literacy and numeracy skills expected of their year level [22]. For this study, NAPLAN scores were converted into binary variables indicating whether children were at/above (0) or below (1) the national minimum standards for their grade level on each test.

### 2.5. Covariates

Covariates (Table 1) were obtained from the linked data, with variables selected a priori based on evidence of an association with OM and/or academic outcomes. Child sex, Aboriginality, birth month/year, percentage of optimum birthweight [23], and gestational age were obtained from the Midwives Notification System (MNS) and Birth Registrations data collections. The WA Genealogical System [24] identified all children born to the same mother between 1974–2012; from this, the number of siblings for each child was calculated. Children with mental health contacts (categorized as yes/no) were identified from the Mental Health Information System (public outpatient contacts) and from HMDC (public and private inpatient contacts) and EDDC (emergency presentations), when a primary mental illness diagnosis (ICD-10-AM ‘F’ codes) was recorded. Birth defects (categorized as present/absent) were identified from the WARDA, which captures details of all birth defects in WA-born children. School readiness scores were obtained from the 2009 Australian Early Development Census (AEDC), a national teacher-reported assessment of children’s performance against 104 items assessing physical, social, emotional, cognitive, and communicative development. Children who score below the 25th percentile (based on national classifications) on any of the five developmental domains are considered developmentally vulnerable or at risk of vulnerability in that area [25]. Child English language status (i.e., whether primary language or not) was also obtained from the AEDC dataset.

Parent age and maternal marital status (both recorded at the time of the child’s birth) were obtained from the MNS and Birth Registrations. The NAPLAN dataset records the highest education level of each parent separately; this information was combined to indicate overall highest education level for both parents.

Area-level socioeconomic and geographic remoteness indices were calculated for the child’s neighborhood of residence at birth. The Index of Relative Socioeconomic Disadvantage (IRSD) [26] was used as the measure of area-level socioeconomic disadvantage. The IRSD is derived from census information capturing factors including income, educational attainment, and unemployment. Neighborhoods are given a score which is categorized into quintiles with 1 representing most disadvantaged and 5 representing least disadvantaged. Under the Australian Statistical Geography Standard [27], geographical areas are classified as metropolitan, inner/outer regional, or remote/very remote based on access to goods and services in an area; for this study, these five categories were collapsed into three (metropolitan, regional, remote).

### 2.6. Statistical Methods

Due to clustering of the data (students within schools), multilevel modelling was conducted. The intra-class correlation coefficient showed that 31%, 37% and 46% of the variance in numeracy, reading, and writing tests, respectively, was explained by unobserved differences between schools, thus indicating the suitability of multilevel modelling. Models grouped children by grade 3 schools (*N* = 830 schools; M = 18 students per school, range 1–99). Some children were missing one (*N* = 435) or two (*N* = 171) of the three NAPLAN test scores; these missing values were imputed with mean school level scores for the corresponding missing test. Missing records for covariates (see counts in Table 1) were handled using multiple imputation (MI). Analyses were conducted using SAS software version 9.4 (SAS Institute Inc., Cary, NC, USA) of the SAS System for Windows [28].

Univariate and multivariate multilevel logistic regressions estimated the odds of children with OM episodes scoring below the national minimum standards on reading, writing, and numeracy tests. Multivariate models included the covariates in Table 1, with the category representing lower risk on each variable coded as the reference group. Analyses were conducted separately for Aboriginal and non-Aboriginal children because of evidence of higher rates of OM incidence and greater severity of disease in Aboriginal populations [2].

Additional models analyzed the effect of (1) age at first OM episode and (2) number of OM episodes on literacy and numeracy scores. Children were categorized into mutually exclusive groups based on age at first OM episode: ≤1 year, 2–3 years, or ≥4 years. For models examining OM episode frequency, all discrete OM episodes were summed for each child. Only month and year of admission/presentation were provided by data custodians; thus, all OM episodes occurring within the same calendar month were combined into a single episode. The additional models were run only for children with at least one OM episode, and with Aboriginal and non-Aboriginal children combined (to achieve statistical power).

## 3. Results

### 3.1. Descriptive Statistics

There were 2666 (13.8%) children with at least one OM episode during the study period. A higher proportion of Aboriginal children had OM episodes (*n* = 256, 17.2%) than non-Aboriginal children (*n* = 2410, 13.6%); this difference was statistically significant, Χ^2^ (1, *N* = 19,262) = 14.9, *p* < 0.001. Children had an average of 1.52 OM episodes during the study period (SD = 1.00; range 1–13; Aboriginal M = 1.62; non-Aboriginal M = 1.50). The median age at first OM episode was 3 years (Aboriginal Mdn = 2 years; non-Aboriginal Mdn = 3 years).

The proportion of children scoring below the benchmark on reading, numeracy, or writing tests was higher for children who had at least one OM episode compared to children with no OM episodes (Table 2). Almost 60% of Aboriginal children with OM episodes scored below the national benchmark on at least one test compared to 11% of non-Aboriginal children with OM episodes.

### 3.2. Logistic Regressions

#### 3.2.1. Any OM Episode

In the unadjusted models, children with at least one OM episode had increased odds of poor performance on all three tests (Table 3). These associations remained statistically significant after adjustment (Table 3). Children with OM episodes had 33%, 35%, and 43% increased odds of scoring below the benchmark on numeracy, reading, and writing, respectively, compared to children without OM episodes. Complete tables of univariate and multivariate models are provided in Supplementary Tables S1 and S2, respectively.

When models were run separately for Aboriginal and non-Aboriginal children, results differed (Table 3). For non-Aboriginal children with OM episodes, there was no statistically significant increase in the odds of poor numeracy or writing performance. However, there was a 31% increase in odds of non-Aboriginal children with an OM episode scoring below the national benchmark on reading, compared to non-Aboriginal children with no OM episodes. Aboriginal children with OM episodes had 70%, 46%, and 79% increased odds of scoring below the benchmark on numeracy, reading, and writing, respectively, when compared to Aboriginal children without OM episodes. A complete table of results is provided in Supplementary Table S3.

#### 3.2.2. Age at First OM Episode and Frequency of OM Episodes

These models were restricted to children with at least one OM episode and combined both Aboriginal and non-Aboriginal children. In the univariate and multivariate models, age at first OM episode and frequency of OM episodes were not significantly associated with scoring below the national benchmark on any NAPLAN test (Table 4).

## 4. Discussion

We found an association between OM secondary care episodes and poorer performance on reading, writing, and numeracy tests, even after adjusting for a range of potential confounding variables. Our findings support previous literature showing an association between OM and poorer educational outcomes [13,14]. Contrary to expectations, however, there was no increased risk associated with earlier age at first OM episode when compared to older age groups. This contrasts with other research [13], and may be related to the use of hospital data, as first secondary care episode may not correlate well with onset of disease. We also did not find that children with repeated OM episodes performed more poorly on literacy and numeracy tests. One other study similarly found no greater risk of poor school performance after multiple OM episodes [29], but at least three other studies did find an association between repeated OM episodes and poorer academic skills [12,30,31]. Further research to clarify these associations are therefore needed. In summary, the results of our study suggest that children who are diagnosed with OM during a secondary care episode may be at risk of poorer academic outcomes, regardless of the timing or frequency of the episodes.

Our results also suggest that OM may be a specific health risk factor for the educational attainment of Aboriginal children. When compared to other Aboriginal children, Aboriginal children with OM episodes had increased odds of up to 79% of not meeting national minimum standards in literacy and numeracy. This is in contrast to an earlier study of 46 Aboriginal children, which found no difference in literacy and reading outcomes among Aboriginal children with and without OM and hearing loss [32]. In our study, Aboriginal children tended to have their first OM episode at a younger age than non-Aboriginal children, and to have more frequent OM episodes. Early onset of OM is one of the strongest predictors of persistent disease [33], and thus an important focus of interventions. Compared to non-Aboriginal children, Aboriginal children with OM have, on average, greater longevity of disease (and thus episodes of poor hearing: 3 months versus 32 months) [34] and may therefore experience greater impacts on speech and language development. OM and its associated hearing loss are treatable conditions, and it is critical that we reduce the prevalence and impact of OM in Aboriginal populations by addressing social and environmental factors that increase OM risk.

In this one-year birth cohort, OM secondary care episodes were relatively common and frequently first experienced prior to age 3. This is concerning, given the potential impact on the development of skills that underlie academic success. Early and effective treatment of conditions that affect hearing and speech, such as OM, is imperative for prevention and/or mitigation of academic issues [34]. Several interventions are suggested. Pneumococcal vaccination is effective at reducing the rates and onset of OM in young populations [35,36], although there is evidence that vaccination is less effective for Aboriginal populations [37]. Tympanostomy tube insertion is effective in preventing repeated episodes of OM [38], which is important due to the association of repeated or persistent OM with hearing loss [6]. However, surgical treatment of OM is less frequently performed for Aboriginal children, despite higher prevalence rates [3]. Antibiotic over-prescription for OM is an issue, but prophylaxis is beneficial for high risk populations, including Aboriginal children [39]. Importantly, preventative measures should address key social determinants that drive the gap in OM rates between Aboriginal and non-Aboriginal populations, and the associated environmental risk factors, including tobacco smoke, poor sanitation, and high-occupancy housing [40]. Such endeavors must include Aboriginal community members in research, intervention design, and service delivery in order to be effective [40,41].

In a pedagogical context, interventions could include: improving teacher awareness of the fluctuating nature of hearing impairment and the impact it can have on learning; protocols to identify students with current or past hearing impairment; improvements to the classroom listening environment; supporting learning through use of non-verbal cues, repetition of instructions, and implementation of predictable routines; and education programs that focus on the development of written and verbal language skills [42]. In regions with high rates of OM, whole-school approaches will likely be needed. Special consideration should be given to children with co-existing special needs (e.g., vision impairment) that may make the impact of hearing loss associated with OM more pronounced [43]. When OM-related hearing problems are detected, educational support plans should involve an interdisciplinary approach involving school nurses, teachers, speech-language therapists, and audiologists [44].

Strengths of this study include the use of linked administrative data, allowing the prospective capture of clinical diagnoses of OM within a large population sample. Use of a standardized test of literacy and numeracy limits bias. Hospital records require clinical diagnoses and are thus less likely than self-report measures to contain reporting bias. However, hospital data can contain selection bias, as members of disadvantaged groups may prefer to attend hospital due to issues with affordability, accessibility, and availability of primary care services [45]. It is possible that some ICD-10-AM codes used to identify OM may be indicative of other disease (e.g., perforation of the tympanic membrane), which may have biased our measure. However, the codes we used are consistent with other published research, the authors of which consulted with ear specialists in defining their inclusion criteria [3]. In addition, OM is primarily managed in the community by general practitioners. As we did not have primary care data our findings represent the more severe end of the clinical spectrum of OM, which may have resulted in larger effect estimates than would be seen if all OM diagnoses were captured. We also did not have data on OM procedures. Tympanostomy tube insertion is conducted to treat persistent OM and to potentially prevent negative developmental consequences [43]; investigations into whether the association between OM and academic attainment is mediated by tympanostomy tube insertion should be conducted. Finally, we did not have information on which children had hearing loss or speech and language difficulties, so a causal link between OM and literacy and numeracy scores cannot be established. Future research should investigate the association between OM-related hearing loss and/or speech and language difficulties and disruptions to learning.

## 5. Conclusions

This study has provided further evidence of an association between OM episodes and literacy and numeracy difficulties in young children. The association was evident even after controlling for a wide range of socio-demographic characteristics and early developmental difficulties. Higher odds of poor literacy and numeracy scores were found regardless of the timing of the first OM episode or the number of OM episodes. Aboriginal children are particularly at risk of both early and repeated episodes of OM, and lower educational attainment associated with OM. Measures to address the social determinants of the high prevalence of OM in Aboriginal populations are urgently needed, in addition to vaccination, surgical treatment, and prophylaxis. Early detection of OM-related hearing problems is also important in order to mitigate impacts on learning.

## Figures and Tables

**Table 1:** Characteristics of the cohort.

		No OM Episodes, % (*n* = 16,596)	≥1 OM Episode ^1^		
Characteristic	Whole Cohort, % (*N* = 19,262)		Overall, % (*n* = 2666)	Aboriginal, % (*n* = 256)	Non-Aboriginal, % (*n* = 2410)
Child sex					
Female ^†^	49.0	49.8	43.5	46.1	43.2
Male	51.0	50.2	56.5	53.9	56.8
Child Indigenous status					
Aboriginal	7.7	7.4	9.6	100	-
Non-Aboriginal ^†^	92.3	92.6	90.4	-	100
Child speaks English as second language					
Yes	6.1	6.2	5.8	22.3	4.1
No ^†^	91.0	91.2	89.9	72.3	91.7
missing	2.9	2.6	4.3	5.5	4.2
Maternal age at cohort member’s birth, y					
<20	5.4	5.4	5.7	23.0	3.8
20–29	43.3	43.0	44.9	56.6	43.7
30–39 ^†^	48.4	48.6	46.8	18.4	49.8
40+	2.9	3.0	2.7	2.0	2.7
Paternal age at cohort member’s birth, y					
<20	1.9	1.9	1.8	7.0	1.2
20–29	30.1	29.8	32.0	35.5	31.6
30–39 ^†^	52.8	53.0	51.4	22.7	54.4
40+	11.1	11.4	9.6	5.5	10.0
missing	4.0	3.8	5.3	29.3	2.7
Maternal marital status at cohort member’s birth					
Married ^†^	90.8	91.0	89.6	67.2	92.0
Never married	7.5	7.3	8.4	25.4	6.6
Divorced/separated/widowed	1.2	1.2	1.3	4.3	1.0
missing	0.5	0.5	0.7	3.1	0.5
Parental highest education level					
University ^†^	28.4	28.5	27.6	6.6	29.9
Vocational	35.6	35.6	35.6	17.2	37.6
High-school	22.5	22.6	22.1	37.9	20.4
missing	13.5	13.3	14.7	38.3	12.2
Total siblings					
0	10.9	10.9	11.1	9.0	11.3
1 ^†^	43.3	43.2	44.0	22.3	46.3
2	28.8	28.8	28.9	24.2	29.4
3 or more	17.0	17.1	16.0	44.5	13.0
Percentage of optimum birthweight ^2^					
Normal (85–114) ^†^	73.2	73.3	72.7	68.0	73.2
Low (< 85)	17.7	17.6	18.2	25.0	17.5
High (< 85)	9.1	9.1	9.0	7.0	9.3
Gestational age					
≥ 37 weeks ^†^	92.7	92.9	91.1	82.0	92.1
33–36 weeks	6.1	6.0	7.0	14.5	6.2
29–32 weeks	0.8	0.7	1.2	2.7	1.0
≤ 28 weeks	0.4	0.4	0.7	0.8	0.7
Season of birth					
Spring	24.5	24.6	24.3	23.8	24.3
Summer ^†^	25.0	24.8	26.1	30.5	25.7
Autumn	26.4	26.5	26.0	25.8	26.0
Winter	24.0	24.1	23.6	19.9	24.0
Any mental health contact					
Yes	1.5	1.3	2.6	6.3	2.2
No ^†^	98.5	98.7	97.4	93.8	97.8
Birth defects					
Yes	5.1	4.6	8.4	5.9	8.6
No ^†^	94.9	95.4	91.6	94.1	91.4
Developmentally vulnerable/at risk (Australian Early Development Census)					
Physical health & wellbeing	9.5	9.1	12.3	25.0	11.0
Social competence	6.8	6.6	8.3	15.6	7.6
Emotional maturity	8.1	7.7	10.3	17.6	9.5
Communication skills & general knowledge	7.1	6.7	9.7	26.6	7.9
Language & cognitive skills (school-based)	10.3	10.0	12.2	34.8	9.8
Area-level socioeconomic disadvantage (Index of Relative Socioeconomic Disadvantage)					
Most disadvantaged	20.6	21.0	18.1	39.8	15.8
2	18.1	18.3	17.4	17.6	17.3
3	18.8	18.7	19.8	13.3	20.5
4	19.6	19.4	20.7	9.8	21.9
Least disadvantaged ^†^	17.1	17.2	16.4	2.3	17.9
missing	5.7	5.4	7.6	17.2	6.6
Geographic remoteness (Australian Statistical Geography Standard)					
Metropolitan ^†^	67.1	66.6	69.9	37.9	73.3
Regional	19.6	19.8	18.3	24.6	17.7
Remote	5.4	5.6	4.3	20.3	2.6
missing	8.0	8.1	7.5	17.2	6.5

Note. OM = Otitis Media. Reference group for Australian Early Development Census is ‘on-track’. ^1^ OM episodes defined as presentations to emergency departments or admissions to hospital where an ICD-10-AM or symptom code indicative of OM was recorded. ^2^ Optimum birthweight is defined by Blair et al. [23] and is based on gestational duration, fetal gender, maternal height, age, and parity. ^†^ Reference group for logistic regressions.

**Table 2:** Proportions of children scoring below the national minimum standards on Grade 3 reading, writing, and numeracy tests.

	NAPLAN Test		
Group	Reading, %	Writing, %	Numeracy, %
Whole Cohort (*N* = 19,262)	6.6	3.5	6.6
No OM episodes (*n* = 16,596)	6.2	3.2	6.3
≥1 OM episode ^1^ (*n* = 2666)	8.8	5.1	8.9
Aboriginal (*N* = 1492)	25.7	21.3	29.2
No OM episodes (*n* = 1236)	24.1	19.4	27.2
≥1 OM episode ^1^ (*n* = 256)	33.2	30.5	39.1
Non-Aboriginal (*N* = 17,770)	5.0	2.0	4.7
No OM episodes (*n* = 15,360)	4.8	1.9	4.6
≥1 OM episode ^1^ (*n* = 2410)	6.2	2.4	5.6

Note. OM = Otitis Media, NAPLAN = National Assessment Program, Literacy and Numeracy. ^1^ OM episodes defined as presentations to emergency departments or admissions to hospital where an ICD-10-AM or symptom code indicative of OM was recorded.

**Table 3:** Univariate and multivariate odds of children scoring below the national benchmarks on grade 3 numeracy, reading and writing tests associated with OM episodes.

	Univariate					
	Reading		Writing		Numeracy	
≥1 OM Episode ^2^	OR (95% CI)	*p*	OR (95% CI)	*p*	OR (95% CI)	*p*
Overall sample	1.48 (1.26 to 1.73)	0.001	1.66 (1.34 to 2.06)	0.001	1.44 (1.23 to 1.70)	0.001
Aboriginal children	1.72 (1.23 to 2.41)	0.001	2.00 (1.42 to 2.82)	0.001	1.82 (1.33 to 2.49)	0.001
Non-Aboriginal children	1.34 (1.11 to 1.61)	0.003	1.31 (0.98 to 1.76)	0.07	1.25 (1.03 to 1.53)	0.030
	**Multivariate ^1^**					
	**Reading**		**Writing**		**Numeracy**	
**≥1 OM Episode ^2^**	**OR (95% CI)**	***p***	**OR (95% CI)**	***p***	**OR (95% CI)**	***p***
Overall sample	1.35 (1.14 to 1.59)	0.001	1.43 (1.13 to 1.81)	0.001	1.33 (1.12 to 1.58)	0.001
Aboriginal children	1.46 (1.02 to 2.09)	0.040	1.79 (1.23 to 2.60)	0.002	1.70 (1.22 to 2.37)	0.002
Non-Aboriginal children	1.31 (1.08 to 1.59)	0.007	1.21 (0.89 to 1.66)	0.22	1.20 (0.98 to 1.47)	0.08

Note. OM = Otitis Media, OR = Odds Ratio, CI = Confidence Interval. ^1^ Adjusted for child sex, English-speaking status, maternal age, paternal age, maternal marital status, parent highest education level, number of siblings, percentage of optimum birthweight, gestational age, season of birth, child mental health contacts, birth defects, developmental vulnerability/risk, neighborhood socioeconomic disadvantage, and neighborhood geographic remoteness. ^2^ OM episodes defined as presentations to emergency departments or admissions to hospital where an ICD-10-AM or symptom code indicative of OM was recorded.

**Table 4:** Univariate and multivariate odds of children scoring below the national benchmarks on Grade 3 numeracy, reading and writing tests associated with age at first OM episode and frequency of OM episodes.

	Univariate					
	Reading		Writing		Numeracy	
≥1 OM Episode ^2^	OR (95% CI)	*p*	OR (95% CI)	*p*	OR (95% CI)	*p*
Age at first OM episode						
≤1 years	0.83 (0.58 to 1.20)	0.32	0.85 (0.52 to 1.38)	0.51	0.99 (0.70 to 1.41)	0.97
2–3 years	0.97 (0.66 to 1.43)	0.89	1.21 (0.74 to 1.98)	0.45	0.84 (0.57 to 1.23)	0.38
≥ 4 years	REF		REF		REF	
Frequency of OM episodes						
Single	REF		REF		REF	
Multiple	1.26 (0.92 to 1.75)	0.15	1.34 (0.88 to 2.06)	0.17	1.09 (0.79 to 1.50)	0.61
	**Multivariate ^1^**					
	**Reading**		**Writing**		**Numeracy**	
**≥1 OM episode^2^**	**OR (95% CI)**	***p***	**OR (95% CI)**	***p***	**OR (95% CI)**	***p***
Age at first OM episode						
≤1 years	0.87 (0.59 to 1.27)	0.46	0.97 (0.56 to 1.68)	0.90	1.02 (0.70 to 1.49)	0.92
2–3 years	1.09 (0.74 to 1.61)	0.65	1.55 (0.89 to 2.68)	0.12	0.94 (0.63 to 1.42)	0.77
≥ 4 years	REF		REF		REF	
Frequency of OM episodes						
Single	REF		REF		REF	
Multiple	1.23 (0.89 to 1.71)	0.21	1.13 (0.70 to 1.80)	0.62	1.01 (0.72 to 1.43)	0.95

Note. Models only include children with OM episodes. OM = Otitis Media, OR = Odds Ratio, CI = Confidence Interval. ^1^ Adjusted for child sex, English-speaking status, maternal age, paternal age, maternal marital status, parent highest education level, number of siblings, percentage of optimum birthweight, gestational age, season of birth, child mental health contacts, birth defects, developmental vulnerability/risk, neighborhood socioeconomic disadvantage, and neighborhood geographic remoteness. ^2^ OM episodes defined as presentations to emergency departments or admissions to hospital where an ICD-10-AM or symptom code indicative of OM was recorded.

## Data Availability

Data used in this project are not publicly available due to ethical and privacy reasons. Other researchers may access the data through the usual application process to the Western Australian Data Linkage Branch and the Western Australian Department of Health Human Research Ethics Committee. Parameters for the data request can be made available on request.

## Supplementary Materials

The following are available online at https://www.mdpi.com/article/10.3390/ijerph182010822/s1, Table S1: Univariate odds of children scoring below the national benchmarks on grade 3 numeracy, reading and writing tests associated with OM episodes and covariates, Table S2: Multivariate odds of children scoring below the national benchmarks on grade 3 numeracy, reading and writing tests associated with OM episodes and covariates, Table S3: Multivariate odds of children scoring below the national benchmarks on grade 3 numeracy, reading and writing tests. Separate models for Aboriginal and non-Aboriginal children.

## Appendix A

Details of OM hospitalizations; number of cases identified from different data sources (Table A1) and number of records identified using different ICD-10-AM or symptom codes, or free text (Table A2).

**Table A1:** Count of children with OM hospitalizations identified from each linked data source.

Data Source	Unique Cases Identified (*N*)
HMDC principal diagnosis	1320
HMDC additional diagnosis	465
EDDC	881
Total	2666

OM = Otitis media, HMDC = Hospital Morbidity Data Collection, EDDC = Emergency Department Data Collection.

**Table A2:** Total counts of records identified using different ICD-10-AM codes, symptom codes, and free text variables.

	*N*	%
ICD-10-AM code		
H65, Non-suppurative otitis media	1783	44.1%
H66, Suppurative and unspecified otitis media	1778	44.0%
H67, Otitis media in diseases classified elsewhere	0	0.0%
H70, Mastoiditis and related conditions	16	0.4%
H71, Cholesteatoma of middle ear	11	0.3%
H72, Perforation of tympanic membrane	165	4.1%
H73, Other disorders of tympanic membrane	13	0.3%
H74, Other disorders of middle ear and mastoid	6	0.1%
H75, Other disorders of middle ear and mastoid in diseases classified elsewhere	0	0.0%
H90.1, Conductive hearing loss, unilateral with unrestricted hearing on the contralateral side	6	0.1%
H90.2, Conductive hearing loss, unspecified	1	0.0%
H90.6, Mixed conductive and sensorineural hearing loss, bilateral	0	0.0%
H90.7, Mixed conductive and sensorineural hearing loss, unilateral with unrestricted hearing on the contralateral side	0	0.0%
H90.8, Mixed conductive and sensorineural hearing loss, unspecified	0	0.0%
H92, Otalgia and effusion of ear	142	3.5%
H95, Postprocedural disorders of ear and mastoid process, not elsewhere classified	0	0.0%
Symptom codes indicating ear discharge	3	0.1%
Free text indicating otitis media and/or ear discharge	117	2.9%
Total	4041	100.0%

ICD-10-AM = International Classification of Diseases, 10th Edition, Australian Modification.
