# Supplementary material for: Children with Secondary Care Episodes for Otitis Media Have Poor Literacy and Numeracy Outcomes: A Data Linkage Study
Source: Int J Environ Res Public Health. 2021 Oct 15;18(20):10822. doi: 10.3390/ijerph182010822 (PMC8535982; doi:10.3390/ijerph182010822)
Supplement: Supplementary file 1 [file ijerph-18-10822-s001.zip › ijerph-1398564-supplementary.pdf]

Supplementary Table S1: Univariate odds of children scoring below the national benchmarks on Grade 3 numeracy, reading and writing tests associated with OM episodes and covariates[a].

	Numeracy	Reading	Writing
	OR (95% CI)	OR (95% CI)	OR (95% CI)
OM episode			
No	REF	REF	REF
Yes	1.44 (1.23 to 1.70)*	1.48 (1.26 to 1.73)*	1.66 (1.34 to 2.06)*
Child sex			
Female	REF	REF	REF
Male	1.04 (0.92 to 1.17)	1.70 (1.50 to 1.93)*	2.40 (2.00 to 2.87)*
Child Indigenous status			
Aboriginal/Torres-Strait Islander	5.99 (5.07 to 7.08)*	4.66 (3.94 to 5.51)*	9.89 (8.06 to 12.14)*
Non-Aboriginal	REF	REF	REF
Child speaks English as second language			
Yes	2.02 (1.60 to 2.56)*	1.67 (1.31 to 2.12)*	2.48 (1.84 to 3.33)*
No	REF	REF	REF
Maternal age at cohort member's birth, y			
<20	1.64 (1.34 to 2.00)*	1.52 (1.24 to 1.87)*	1.98 (1.54 to 2.54)*
20-29	REF	REF	REF
30-39	0.59 (0.51 to 0.68)*	0.58 (0.50 to 0.67)*	0.55 (0.45 to 0.67)*
40+	0.69 (0.45 to 1.06)	0.67 (0.44 to 1.01)	0.42 (0.21 to 0.85)*
Paternal age at cohort member's birth, y			
<20	1.82 (1.22 to 2.72)*	1.71 (1.20 to 2.43)*	2.16 (1.42 to 3.27)*
20-29	REF	REF	REF
30-39	0.63 (0.55 to 0.73)*	0.63 (0.54 to 0.72)*	0.55 (0.45 to 0.68)*
40+	0.55 (0.43 to 0.71)*	0.63 (0.49 to 0.81)*	0.49 (0.34 to 0.69)*
Maternal marital status at cohort member's birth			
Married	REF	REF	REF
Unmarried	1.65 (1.37 to 2.00)*	2.02 (1.69 to 2.42)*	3.08 (2.47 to 3.82)*
Divorced/separated/widowed	2.22 (1.46 to 3.37)*	1.74 (1.09 to 2.77)*	2.43 (1.37 to 4.31)*
Parental highest education level			
University	REF	REF	REF
Vocational	2.88 (2.28 to 3.63)*	2.69 (2.14 to 3.38)*	2.98 (2.06 to 4.32)*
High-school	5.35 (4.23 to 6.77)*	4.90 (3.91 to 6.14)*	8.06 (5.44 to 11.94)*
Total siblings			
0	1.32 (1.07 to 1.63)*	1.27 (1.03 to 1.57)*	1.33 (0.98 to 1.79)
1	REF	REF	REF
2	1.10 (0.94 to 1.29)	1.21 (1.03 to 1.41)*	1.26 (1.00 to 1.57)*
3 or more	2.04 (1.74 to 2.40)*	2.08 (1.77 to 2.44)*	2.58 (2.07 to 3.20)*
Percentage of optimum birthweight			
Normal (85-114%)	REF	REF	REF
Low (< 85%)	1.42 (1.23 to 1.65)*	1.37 (1.19 to 1.59)*	1.60 (1.32 to 1.95)*
High (≥ 115%)	0.92 (0.73 to 1.16)	0.83 (0.65 to 1.05)	0.80 (0.57 to 1.12)
Gestational age			
≥ 37 weeks	REF	REF	REF
33-36 weeks	1.30 (1.03 to 1.64)*	1.25 (0.99 to 1.58)	1.19 (0.86 to 1.63)
29-32 weeks	2.62 (1.57 to 4.39)*	1.71 (0.96 to 3.02)	3.20 (1.70 to 6.05)*
≤ 28 weeks	2.07 (1.01 to 4.24)*	1.12 (0.47 to 2.62)	0.44 (0.10 to 1.93)
Season of birth			
Spring	REF	REF	REF

Summer	1.17 (0.98 to 1.40)	1.22 (1.02 to 1.45)*	0.93 (0.72 to 1.20)
Autumn	1.40 (1.18 to 1.67)*	1.38 (1.16 to 1.64)*	1.48 (1.18 to 1.87)*
Winter	1.01 (0.84 to 1.21)	1.11 (0.93 to 1.33)	1.13 (0.88 to 1.46)
Any mental health contact			
Yes	2.48 (1.72 to 3.58)*	2.09 (1.43 to 3.07)*	4.63 (3.07 to 6.98)*
No	REF	REF	REF
Birth defects			
Yes	1.19 (0.91 to 1.56)	0.95 (0.71 to 1.27)	1.20 (0.84 to 1.73)
No	REF	REF	REF
Developmentally vulnerable/at-risk (Australian Early Development Census)[b]			
Physical health & wellbeing	3.14 (2.68 to 3.68)*	3.00 (2.56 to 3.51)*	4.12 (3.37 to 5.04)*
Social competence	3.68 (3.09 to 4.39)*	3.36 (2.82 to 4.00)*	5.56 (4.47 to 6.92)*
Emotional maturity	2.65 (2.23 to 3.15)*	2.66 (2.25 to 3.15)*	4.09 (3.31 to 5.04)*
Communication skills & general knowledge	4.13 (3.49 to 4.89)*	4.12 (3.49 to 4.87)*	5.64 (4.56 to 6.97)*
Language & cognitive skills (school based)	6.45 (5.59 to 7.45)*	5.25 (4.55 to 6.06)*	8.09 (6.71 to 9.76)*
Area-level socioeconomic disadvantage			
Most disadvantaged	3.71 (2.74 to 5.04)*	3.84 (2.93 to 5.04)*	4.28 (2.81 to 6.52)*
2	2.79 (2.06 to 3.79)*	2.81 (2.13 to 3.71)*	3.19 (2.11 to 4.83)*
3	2.29 (1.64 to 3.18)*	2.38 (1.79 to 3.16)*	1.77 (1.14 to 2.74)*
4	1.64 (1.20 to 2.25)*	1.41 (1.05 to 1.89)*	1.56 (1.01 to 2.41)*
Least disadvantaged	REF	REF	REF
Geographic remoteness			
Metropolitan	REF	REF	REF
Regional	1.42 (1.18 to 1.69)*	1.40 (1.14 to 1.72)*	1.54 (1.19 to 1.99)*
Remote	2.39 (1.80 to 3.18)*	2.84 (2.19 to 3.70)*	4.46 (3.07 to 6.47)*

OM=Otitis Media, REF= Reference category, OR= Odds Ratio, CI = Confidence Interval

[a] All variables were entered into separate models. [b] Reference category for all Australian Early Development Census Domains is 'on-track'.

**p*<.05

Supplementary Table S2: Multivariate odds of children scoring below the national benchmarks on Grade 3 numeracy, reading and writing tests associated with OM episodes and covariates.

	Numeracy	Reading	Writing
	OR (95% CI)	OR (95% CI)	OR (95% CI)
OM episode			
No	REF	REF	REF
Yes	1.33 (1.12 to 1.58)*	1.35 (1.14 to 1.59)*	1.43 (1.13 to 1.81)*
Child sex			
Female	REF	REF	REF
Male	0.88 (0.77 to 1.00)	1.54 (1.35 to 1.75)*	2.16 (1.77 to 2.62)*
Child Indigenous status			
Aboriginal/Torres-Strait Islander	2.94 (2.44 to 3.54)*	2.18 (1.81 to 2.63)*	3.63 (2.85 to 4.63)*
Non-Aboriginal	REF	REF	REF
Child speaks English as second language			
Yes	1.23 (0.96 to 1.58)	0.95 (0.74 to 1.23)	1.22 (0.87 to 1.72)
No	REF	REF	REF
Maternal age at cohort member's birth, y			
<20	1.64 (1.34 to 2.00)*	1.52 (1.24 to 1.87)*	1.98 (1.54 to 2.54)*
20-29	REF	REF	REF
30-39	0.82 (0.69 to 0.98)*	0.81 (0.68 to 0.96)*	0.87 (0.68 to 1.11)
40+	0.86 (0.53 to 1.38)	0.82 (0.52 to 1.30)	0.64 (0.30 to 1.37)
Paternal age at cohort member's birth, y			
<20	1.23 (0.79 to 1.92)	1.14 (0.75 to 1.74)	1.27 (0.75 to 2.15)
20-29	REF	REF	REF
30-39	0.90 (0.75 to 1.07)	0.91 (0.76 to 1.08)	0.88 (0.69 to 1.12)
40+	0.83 (0.62 to 1.11)	0.99 (0.74 to 1.33)	0.94 (0.62 to 1.41)
Maternal marital status at cohort member's birth			
Married	REF	REF	REF
Unmarried	0.88 (0.71 to 1.08)	1.21 (0.99 to 1.49)	1.64 (1.27 to 2.11)*
Divorced/separated/ widowed	1.56 (1.01 to 2.42)*	1.20 (0.75 to 1.90)	1.56 (0.86 to 2.82)
Parental highest education level			
University	REF	REF	REF
Vocational	2.17 (1.72 to 2.74)*	2.01 (1.60 to 2.52)*	1.99 (1.31 to 3.01)*
High-school	2.79 (2.18 to 3.59)*	2.59 (2.03 to 3.29)*	3.29 (2.10 to 5.13)*
Total siblings			
0	1.11 (0.88 to 1.38)	1.06 (0.85 to 1.33)	1.00 (0.71 to 1.39)
1	REF	REF	REF
2	1.01 (0.85 to 1.19)	1.11 (0.94 to 1.3)	1.12 (0.88 to 1.43)
3 or more	1.38 (1.16 to 1.64)*	1.47 (1.24 to 1.74)*	1.66 (1.31 to 2.12)*
Percentage of optimum birthweight			
Normal (85-114%)	REF	REF	REF
Low (< 85%)	1.31 (1.12 to 1.54)*	1.30 (1.12 to 1.52)*	1.45 (1.17 to 1.80)*
High (≥ 115%)	1.02 (0.80 to 1.30)	0.90 (0.71 to 1.15)	0.99 (0.70 to 1.42)
Gestational age			
≥ 37 weeks	REF	REF	REF
33-36 weeks	1.11 (0.86 to 1.42)	1.09 (0.86 to 1.39)	0.91 (0.64 to 1.30)
29-32 weeks	1.51 (0.86 to 2.64)	1.00 (0.55 to 1.83)	1.70 (0.85 to 3.40)
≤ 28 weeks	1.61 (0.75 to 3.46)	0.97 (0.40 to 2.34)	0.39 (0.08 to 1.80)
Season of birth			

Spring	REF	REF	REF
Summer	1.13 (0.94 to 1.36)	1.18 (0.98 to 1.41)	0.91 (0.70 to 1.20)
Autumn	1.25 (1.04 to 1.50)*	1.24 (1.04 to 1.49)*	1.34 (1.04 to 1.73)*
Winter	1.03 (0.85 to 1.25)	1.13 (0.93 to 1.36)	1.19 (0.91 to 1.55)
Any mental health contact			
Yes	1.68 (1.13 to 2.48)*	1.33 (0.88 to 2.00)	2.98 (1.90 to 4.67)*
No	REF	REF	REF
Birth defects			
Yes	1.16 (0.88 to 1.55)	0.85 (0.63 to 1.15)	1.06 (0.71 to 1.58)
No	REF	REF	REF
Developmentally vulnerable/at-risk (Australian Early Development Census)[a]			
Physical health & wellbeing	1.26 (1.04 to 1.53)*	1.26 (1.04 to 1.51)*	1.28 (0.99 to 1.64)
Social competence	1.34 (1.06 to 1.71)*	1.16 (0.92 to 1.47)	1.45 (1.07 to 1.96)*
Emotional maturity	1.14 (0.91 to 1.43)	1.20 (0.97 to 1.49)	1.33 (1.01 to 1.75)*
Communication skills & general knowledge	1.23 (1.00 to 1.53)	1.42 (1.15 to 1.75)*	1.39 (1.05 to 1.83)*
Language & cognitive skills (school based)	3.42 (2.87 to 4.07)*	2.53 (2.13 to 3.01)*	2.88 (2.28 to 3.63)*
Area-level socioeconomic disadvantage			
Most disadvantaged	1.94 (1.42 to 2.67)*	2.11 (1.6 to 2.79)*	1.71 (1.10 to 2.65)*
2	1.72 (1.26 to 2.34)*	1.70 (1.29 to 2.25)*	1.47 (0.96 to 2.26)
3	1.63 (1.15 to 2.31)*	1.69 (1.27 to 2.25)*	1.03 (0.66 to 1.60)
4	1.26 (0.91 to 1.76)	1.08 (0.80 to 1.45)	0.97 (0.61 to 1.54)
Least disadvantaged	REF	REF	REF
Geographic remoteness			
Metropolitan	REF	REF	REF
Regional	1.21 (1.02 to 1.43)*	1.16 (0.96 to 1.39)	1.25 (0.98 to 1.60)
Remote	1.67 (1.27 to 2.22)*	2.37 (1.87 to 3.00)*	3.10 (2.27 to 4.25)*

OM=Otitis Media, REF= Reference category, OR= Odds Ratio, CI = Confidence Interval

[a] Reference category for all Australian Early Development Census Domains is 'on-track'.

*p<.05

Supplementary Table S3: Multivariate odds of children scoring below the national benchmarks on Grade 3 numeracy, reading and writing tests. Separate models for Aboriginal and non-Aboriginal children.

	Numeracy		Reading		Writing	
	OR (95% CI)		OR (95% CI)		OR (95% CI)	
	Aboriginal	Non-Aboriginal	Aboriginal	Non-Aboriginal	Aboriginal	Non-Aboriginal
OM episode						
No	REF	REF	REF	REF	REF	REF
Yes	1.70 (1.22 to 2.37)*	1.20 (0.98 to 1.47)	1.46 (1.02 to 2.09)*	1.31 (1.08 to 1.59)*	1.79 (1.23 to 2.60)*	1.21 (0.89 to 1.66)
Child sex						
Female	REF	REF	REF	REF	REF	REF
Male	0.86 (0.66 to 1.12)	0.88 (0.76 to 1.03)	1.96 (1.47 to 2.61)*	1.45 (1.25 to 1.68)*	2.31 (1.69 to 3.16)*	2.16 (1.67 to 2.80)*
Child speaks English as second language						
Yes	1.83 (1.23 to 2.71)*	0.78 (0.55 to 1.11)	1.38 (0.89 to 2.14)	0.70 (0.47 to 1.03)*	1.70 (0.99 to 2.90)	0.58 (0.30 to 1.09)
No	REF	REF	REF	REF	REF	REF
Maternal age at cohort member's birth, y						
<20	0.85 (0.56 to 1.29)	1.09 (0.76 to 1.57)	0.98 (0.63 to 1.54)	0.83 (0.58 to 1.18)	0.80 (0.51 to 1.28)	0.93 (0.55 to 1.56)
20-29	REF	REF	REF	REF	REF	REF
30-39	0.79 (0.53 to 1.19)	0.83 (0.69 to 1.01)	0.86 (0.53 to 1.37)	0.81 (0.67 to 0.97)*	0.89 (0.56 to 1.44)	0.89 (0.65 to 1.21)
40+	0.67 (0.15 to 2.92)	0.87 (0.52 to 1.45)	0.51 (0.09 to 2.86)	0.82 (0.50 to 1.33)	2.23 (0.50 to 9.98)	0.38 (0.14 to 1.04)
Paternal age at cohort member's birth, y						
<20	1.64 (0.85 to 3.15)	0.89 (0.48 to 1.65)	1.12 (0.60 to 2.11)	1.09 (0.64 to 1.88)	1.81 (0.98 to 3.35)	0.76 (0.34 to 1.71)
20-29	REF	REF	REF	REF	REF	REF
30-39	0.86 (0.59 to 1.24)	0.95 (0.78 to 1.16)	0.91 (0.53 to 1.55)	0.94 (0.77 to 1.14)	1.24 (0.78 to 1.98)	0.82 (0.60 to 1.12)
40+	0.72 (0.34 to 1.54)	0.89 (0.64 to 1.23)	0.60 (0.23 to 1.53)	1.10 (0.80 to 1.50)	1.24 (0.78 to 1.98)	1.19 (0.72 to 1.96)
Maternal marital status at cohort member's birth						
Married	REF	REF	REF	REF	REF	REF
Unmarried	1.01 (0.73 to 1.40)	0.84 (0.64 to 1.11)	1.13 (0.80 to 1.61)	1.26 (0.98 to 1.63)	1.20 (0.83 to 1.73)	2.13 (1.52 to 2.98)*
Divorced/separated/ widowed	1.51 (0.68 to 3.32)	1.55 (0.92 to 2.63)	1.02 (0.43 to 2.39)	1.24 (0.71 to 2.15)	1.34 (0.55 to 3.25)	1.69 (0.77 to 3.74)
Parental highest education level						
University	REF	REF	REF	REF	REF	REF
Vocational	1.11 (0.42 to 2.94)	2.37 (1.81 to 3.10)*	1.42 (0.58 to 3.52)	2.06 (1.62 to 2.61)*	1.46 (0.51 to 4.20)	2.07 (1.28 to 3.36)*
High-school	1.89 (0.98 to 3.62)	2.84 (2.16 to 3.72)*	1.62 (0.77 to 3.42)	2.76 (2.15 to 3.53)*	3.12 (1.10 to 8.88)*	2.95 (1.73 to 5.03)*

	Numeracy		Reading		Writing	
	OR (95% CI)		OR (95% CI)		OR (95% CI)	
	Aboriginal	Non-Aboriginal	Aboriginal	Non-Aboriginal	Aboriginal	Non-Aboriginal
Total siblings						
0	1.04 (0.62 to 1.75)	1.15 (0.89 to 1.47)	1.77 (1.02 to 3.07)*	0.94 (0.73 to 1.22)	1.26 (0.70 to 2.25)	0.87 (0.57 to 1.33)
1	REF	REF	REF	REF	REF	REF
2	1.30 (0.87 to 1.93)	0.96 (0.80 to 1.16)	1.59 (1.03 to 2.45)*	1.04 (0.87 to 1.24)	1.14 (0.72 to 1.79)	1.11 (0.83 to 1.48)
3 or more	1.71 (1.19 to 2.45)*	1.31 (1.07 to 1.61)*	1.87 (1.26 to 2.78)*	1.45 (1.20 to 1.76)*	1.33 (0.88 to 2.02)	1.96 (1.45 to 2.64)*
Percentage of optimum birthweight						
Normal (85-114%)	REF	REF	REF	REF	REF	REF
Low (< 85%)	1.72 (1.28 to 2.32)*	1.17 (0.96 to 1.41)	1.23 (0.89 to 1.70)	1.33 (1.11 to 1.59)*	1.48 (1.05 to 2.08)*	1.44 (1.09 to 1.9)*
High (≥ 115%)	1.26 (0.74 to 2.15)	0.97 (0.74 to 1.27)	0.98 (0.54 to 1.76)	0.88 (0.67 to 1.16)	1.42 (0.78 to 2.61)	0.84 (0.53 to 1.33)
Gestational age						
≥ 37 weeks	REF	REF	REF	REF	REF	REF
33-36 weeks	1.09 (0.69 to 1.70)	1.12 (0.83 to 1.51)	1.25 (0.77 to 2.01)	1.06 (0.79 to 1.41)	†	0.90 (0.56 to 1.46)
29-32 weeks	0.89 (0.32 to 2.47)	1.77 (0.92 to 3.40)	1.33 (0.45 to 3.96)	0.79 (0.36 to 1.73)	†	1.40 (0.55 to 3.56)
≤ 28 weeks	0.61 (0.14 to 2.71)	2.65 (1.14 to 6.15)*	2.04 (0.48 to 8.62)	0.77 (0.23 to 2.61)	†	1.11 (0.23 to 5.45)
Season of birth						
Spring	REF	REF	REF	REF	REF	REF
Summer	1.02 (0.70 to 1.47)	1.18 (0.95 to 1.47)	0.79 (0.53 to 1.16)	1.32 (1.07 to 1.64)*	0.97 (0.63 to 1.50)	0.87 (0.61 to 1.23)
Autumn	1.22 (0.84 to 1.75)	1.27 (1.03 to 1.57)*	0.87 (0.59 to 1.29)	1.40 (1.14 to 1.72)*	1.57 (1.03 to 2.40)*	1.22 (0.89 to 1.67)
Winter	1.05 (0.72 to 1.55)	1.07 (0.85 to 1.34)	0.82 (0.55 to 1.24)	1.27 (1.02 to 1.58)*	1.37 (0.87 to 2.15)	1.14 (0.81 to 1.60)
Any mental health contact						
Yes	0.97 (0.46 to 2.04)	1.98 (1.26 to 3.10)*	0.83 (0.37 to 1.89)	1.60 (1.01 to 2.53)*	2.53 (1.19 to 5.41)*	3.23 (1.86 to 5.61)*
No	REF	REF	REF	REF	REF	REF
Birth defects						
Yes	0.90 (0.45 to 1.84)	1.24 (0.91 to 1.69)	0.70 (0.32 to 1.53)	0.91 (0.66 to 1.26)	0.98 (0.44 to 2.18)	1.18 (0.75 to 1.88)
No	REF	REF	REF	REF	REF	REF
Developmentally vulnerable/at-risk (Australian Early Development Census)[a]						
Physical health & wellbeing	0.94 (0.66 to 1.36)	1.36 (1.09 to 1.71)*	0.86 (0.58 to 1.28)	1.38 (1.11 to 1.71)	1.16 (0.77 to 1.74)	1.24 (0.90 to 1.71)
Social competence	1.36 (0.87 to 2.11)	1.39 (1.04 to 1.85)*	1.08 (0.67 to 1.74)	1.20 (0.91 to 1.59)	1.47 (0.91 to 2.39)	1.44 (0.98 to 2.12)

	Numeracy		Reading		Writing	
	OR (95% CI)		OR (95% CI)		OR (95% CI)	
	Aboriginal	Non-Aboriginal	Aboriginal	Non-Aboriginal	Aboriginal	Non-Aboriginal
Emotional maturity	1.39 (0.93 to 2.09)	1.04 (0.79 to 1.36)	1.49 (0.97 to 2.30)	1.11 (0.86 to 1.43)	1.20 (0.77 to 1.89)	1.33 (0.93 to 1.91)
Communication skills & general knowledge	1.16 (0.78 to 1.75)	1.30 (1.01 to 1.67)*	2.32 (1.49 to 3.62)*	1.21 (0.94 to 1.55)	1.44 (0.91 to 2.28)	1.41 (0.99 to 2.00)
Language & cognitive skills (school based)	2.47 (1.79 to 3.41)*	3.99 (3.25 to 4.9)*	1.70 (1.19 to 2.42)*	3.04 (2.49, 3.71)*	1.75 (1.21, 2.52)*	4.16 (3.10, 5.58)*
Area-level socioeconomic disadvantage						
Most disadvantaged	1.09 (0.49 to 2.43)	2.15 (1.53 to 3.02)*	1.30 (0.52 to 3.22)	2.25 (1.67, 3.04)*	1.26 (0.53, 3.02)	1.72 (0.98, 3.00)
2	1.22 (0.55 to 2.70)	1.72 (1.22 to 2.41)*	0.89 (0.37 to 2.12)	1.87 (1.37, 2.54)*	1.11 (0.46, 2.72)	1.47 (0.85, 2.53)
3	0.78 (0.33 to 1.83)	1.80 (1.25 to 2.58)*	1.24 (0.49 to 3.13)	1.74 (1.27, 2.37)*	1.11 (0.46, 2.72)	0.95 (0.54, 1.70)
4	0.83 (0.35 to 1.93)	1.32 (0.93 to 1.88)	0.57 (0.21 to 1.55)	1.17 (0.84, 1.63)	0.84 (0.31, 2.25)	0.95 (0.55, 1.64)
Least disadvantaged	REF	REF	REF	REF	REF	REF
Geographic remoteness						
Metropolitan	REF	REF	REF	REF	REF	REF
Regional	1.29 (0.89 to 1.87)	1.16 (0.95 to 1.43)	1.08 (0.70 to 1.66)	1.14 (0.93, 1.39)	1.19 (0.76, 1.88)	1.26 (0.93, 1.70)
Remote	2.03 (1.38 to 2.98)*	1.06 (0.70 to 1.59)	2.44 (1.49 to 3.99)*	2.03 (1.49, 2.76)*	3.35 (2.02, 5.54)*	1.95 (1.17, 3.25)*

Note. OM=Otitis Media, REF= Reference category, OR= Odds Ratio, CI = Confidence Interval

[a] Reference category for all Australian Early Development Census Domains is ‘on-track’

*$p<0.05$

†values omitted due to small cell sizes
